# Phase II proof‐of‐concept study of atorvastatin in castration‐resistant prostate cancer

**DOI:** 10.1111/bju.15851

**Published:** 2022-08-12

**Authors:** Linda K. Rushworth, Carolyn Loveridge, Mark Salji, Martin MacLeod, Ernest Mui, David Sumpton, Matthew Neilson, Ann Hedley, Laura Alexander, Elaine McCartney, Rachana Patel, Jan Wallace, Christian Delles, Rob Jones, Hing Y. Leung

**Affiliations:** ^1^ Institute of Cancer Sciences, College of Medical, Veterinary and Life Sciences University of Glasgow Glasgow UK; ^2^ CRUK Beatson Institute Glasgow UK; ^3^ Beatson West of Scotland Cancer Centre Glasgow UK; ^4^ CRUK West of Scotland Clinical Trials Unit Glasgow UK; ^5^ Institute of Cardiovascular and Medical Sciences, College of Medical, Veterinary and Life Sciences University of Glasgow Glasgow UK

**Keywords:** atorvastatin, statins, cholesterol, castration resistant prostate cancer, prostate specific antigen, #PCSM, #ProstateCancer, #uroonc

## Abstract

**Objectives:**

To test for evidence of statin‐mediated effects in patients with castration‐resistant prostate cancer (CRPC) as post‐diagnosis use of statins in patients with prostate cancer is associated with favourable survival outcome.

**Patients and Methods:**

The SPECTRE trial was a 6‐weeks‐long proof‐of‐concept single‐arm Phase II treatment trial, combining atorvastatin and androgen deprivation therapy in patients with CRPC (regardless of metastatic status), designed to test for evidence of statin‐mediated effects in patients with CRPC. The primary study endpoint was the proportion of patients achieving a ≥50% drop from baseline in prostate‐specific antigen (PSA) levels at any time over the 6‐week period of atorvastatin medication (PSA response). Exploratory endpoints include PSA velocity and serum metabolites identified by mass spectrometry .

**Results:**

At the scheduled interim analysis, one of 12 patients experienced a ≥50% drop in PSA levels (primary endpoint), with ≥2 patients satisfying the primary endpoint required for further recruitment. All 12 patients experienced substantial falls in serum cholesterol levels following statin treatment. While all patients had comparable pre‐study PSA velocities, six of 12 patients showed decreased PSA velocities after statin treatment, suggestive of disease stabilization. Unbiased metabolomics analysis on serial weekly blood samples identified tryptophan to be the dominant metabolite associated with patient response to statin.

**Conclusions:**

Data from the SPECTRE study provide the first evidence of statin‐mediated effects on CRPC and early sign of disease stabilization. Our data also highlight the possibility of altered tryptophan metabolism being associated with tumour response.

## Introduction

Prostate cancer is the second most common cause of cancer deaths in men in the Western world [1]. For advanced disease, androgen deprivation therapy (ADT) remains the first‐line hormonal treatment option, with docetaxel and cabazitaxel the first‐ and second‐line chemotherapy treatments, respectively [2, 3]. Upfront chemotherapy in combination with ADT gives a robust survival benefit [3, 4]. Similarly, upfront enzalutamide along with testosterone suppression, with or without early docetaxel, improved survival in men with metastatic, hormone‐sensitive prostate cancer [5]. Health‐related quality‐of‐life analysis revealed that overall health and quality of life were maintained in the enzalutamide group despite initial worsening of self‐reported fatigue, cognitive function, and physical function [6]. Recent published data on triple systemic therapy (ADT, docetaxel, and a second‐generation androgen signalling inhibitor [either abiraterone with prednisone or darolutamide]) improved overall survival in patients with *de novo* metastatic castrate‐sensitive prostate cancer, without excessively increasing toxicity [7, 8]. However, despite these novel combination regimes, >25% of patients with metastatic prostate cancer still die within 5 years of diagnosis.

The role of statins in prostate carcinogenesis and patient outcome is hotly debated. While there is significant controversy concerning whether statins might reduce the overall risk of prostate cancer, men taking statins for at least 3 years post diagnosis of non‐metastatic prostate cancer have 39% lower cancer mortality rates and a 23% lower chance of developing distant metastases [9]. Individuals with elevated total serum cholesterol and high‐density lipoprotein were at increased risk of developing aggressive prostate cancer [10]. Recent *in vitro* and *in vivo* preclinical research further implicated altered cancer cholesterol metabolism in progressive disease, with statins enhancing tumour response to ADT [11, 12]. Specifically, in a preclinical model of CRPC, we recently demonstrated evidence of subclinical cachexia, whereby host adipose tissues are mobilized to form free fatty acids (FFAs) which further support enhanced hepatic cholesterol synthesis. Within the tumour microenvironment, CRPC upregulates the receptor for cholesterol uptake to increase cholesterol availability in tumour cells, thus supporting *de novo* androgen biosynthesis to overcome ADT [11].

Statins are currently licensed for and widely used in the treatment of hypercholesterolaemia and the prevention of cardiovascular disease by inhibiting the function of 3‐hydroxy‐3‐methylglutaryl‐CoA (HMG CoA) reductase, the rate‐controlling enzyme of the mevalonate pathway required for cholesterol synthesis. Statins are reported to reduce PSA levels via enhanced degradation of the androgen receptor protein [13, 14]. Of note, adding simvastatin to ADT in human LNCaP (androgen receptor‐positive and hormone‐dependent) prostate cancer cells resulted in enhanced growth inhibition [15]. However, to date, no trials have been carried out to test if a short course of statin treatment mediated effects in clinical CRPC. The SPECTRE (Combined Suppression of Cholesterol Bioavailability and Androgen Deprivation Therapy to Treat Castration‐Resistant Prostate Cancer) trial is a single‐arm phase II trial in patients with CRPC, conducted to investigate evidence of statin‐mediated impact on CRPC on combined ADT with suppression of cholesterol bioavailability using atorvastatin. Here, we present findings from the interim analysis of Stage 1 of the SPECTRE study, which did not progress to Stage 2 (see Trial Design in the Methods Section for details).

## Patients and Methods

### Trial Design

**Fig. 1:**
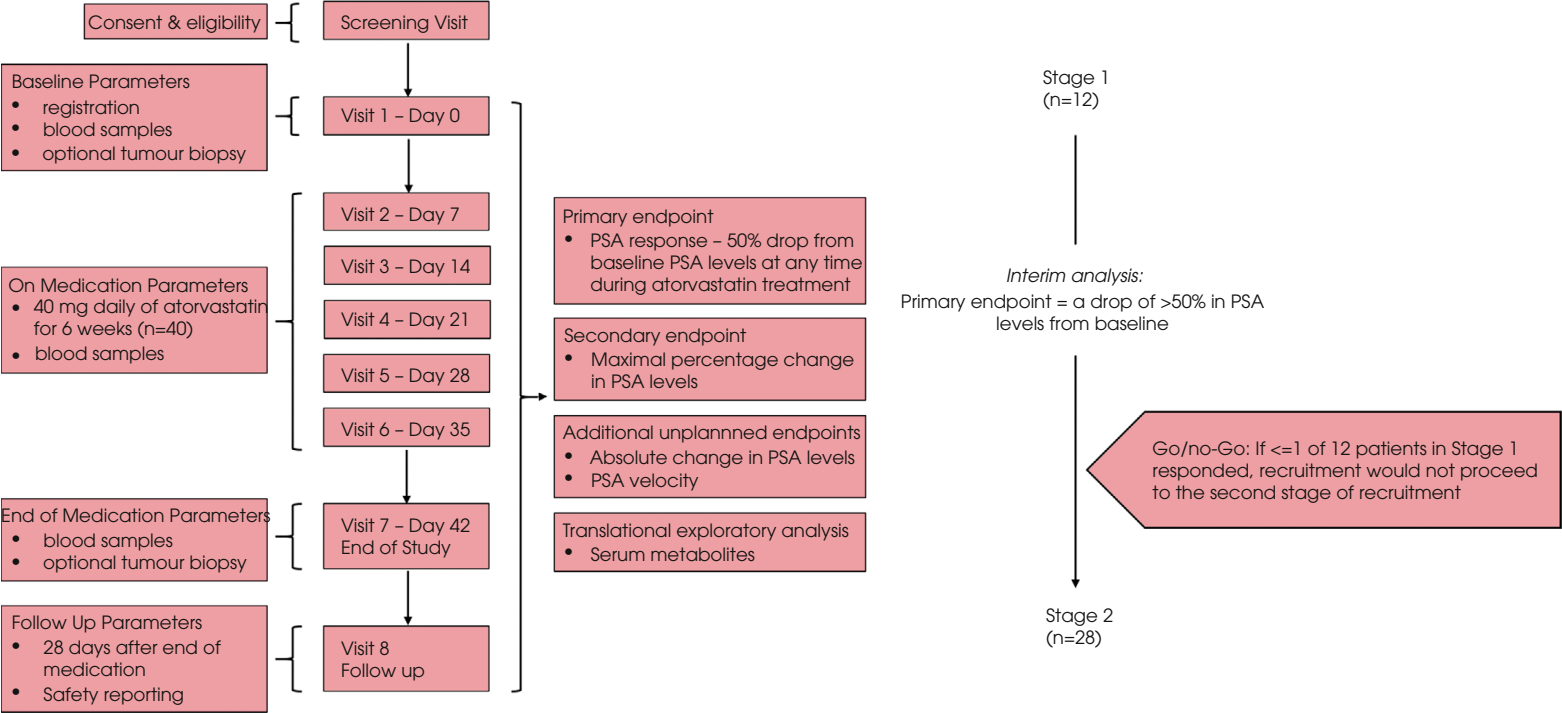
Schematic of trial workflow. Left panel: Overall study design along with details of study parameters (before, during and after atorvastatin treatment) and study endpoints. Right panel: The SPECTRE study was designed (at 90% power, 10% 1‐sided significance level) to distinguish an ‘ineffective’ PSA response rate of ≤10% from an ‘effective’ PSA response rate of ≥30%. This required 12 patients to be recruited at the first stage. If ≤1 of these 12 patients responded, recruitment would not proceed to the second stage, which would require a total of 35 evaluable patients (with 40 as the recruitment target; Appendix S1).

The trial was conducted at the Beatson West of Scotland Cancer Centre. All patients provided written informed consent. SPECTRE was a single arm Phase II study which used a Simon two‐stage optimal design (90% power, 10% one‐sided significance level) to distinguish an ‘ineffective’ PSA response rate of ≤10% from an ‘effective’ PSA response rate of ≥30% (Fig. 1). This required 12 evaluable patients to be recruited at the first stage. If ≤1 of these 12 patients responded, recruitment would not proceed to the second stage of recruitment. If the study continued to the second stage, a further 23 evaluable patients would be recruited (total of 35), with the study aiming to recruit 40 patients in total. A patient was classified as non‐evaluable if they did not complete 80% of 6 weeks of atorvastatin medication and had neither a drop of ≥50% in PSA levels from baseline nor PSA progression. Patients were also considered as non‐evaluable if only three PSA measurements were available after start of atorvastatin medication and none of these corresponded to a ≥50% drop from baseline or PSA progression.

### Patients and Study Medication

Eligible patients were 18 years old or over, with proven adenocarcinoma of the prostate, defined as: histological or cytological evidence of prostate cancer, or PSA >100 ng/mL at time of diagnosis and presence of more than four bone metastases. Patients were required to have disease progression despite ongoing castration therapy (either using LHRH analogue or prior surgical orchiectomy, with or without abiraterone or enzalutamide) as judged by rising serial PSA measurements, regardless of metastatic status. This was based on a series of at least three readings each taken at least 7 days apart. The third reading was required to be ≥2 ng/mL. Patients who received prior bicalutamide, flutamide or nilutamide had to have proven PSA progression after withdrawal of the drug. Castrate levels of serum testosterone (<1.7 nmoL/L) were required. Patients could not have had therapy with statins or other cholesterol‐lowering drug during a 28‐day period prior to the initiation of trial medication. A screening visit was carried out (Day −28 to Day 1) during which informed consent and medical history were obtained, as well as biochemical tests. Following commencement of treatment, atorvastatin was administered orally, at a dose of 40 mg each day, for a total of 6 weeks. Any adverse effects were recorded, and serious adverse effects immediately reported to the Pharmacovigilance Office, CRUK Clinical Trials Unit.

### Study Endpoints

The primary study endpoint was the proportion of patients achieving ≥50% drop from baseline in PSA levels at any time over the 6‐week period of atorvastatin medication (PSA response). PSA levels were measured weekly during this period. The secondary endpoint was maximal percentage change in PSA levels. Additional (exploratory) analyses were performed to study absolute change in serial PSA levels during treatment and PSA velocities before and after statin treatment.

Based on serial serum PSA levels obtained during and at the conclusion of the study, the maximum absolute drop in PSA (in ng/mL) was determined as previously reported [16] and presented as a waterfall plot. The methods of exact inference described in Koyama and Chen [17] were used to estimate the proportion of patients with PSA response and the associated 80% CI.

### Serum Measurements

Serum measurements for the following tests were taken at the beginning of treatment (baseline) and weekly until the end of treatment and performed according to local standard procedures: standard of care clinical assays for PSA, testosterone, lipid profiling, and serum biochemistry. Serum FFA levels were measured using the Free Fatty Acid Assay Kit (Abcam, Cambridge, UK) as per the manufacturer's instructions. For clinical reasons, serum samples for biochemical analysis were not available for Patient 015, so FFA levels were not measured for this patient.

### Data Analysis

Data plotting and statistical analyses including two‐way ANOVA with Sidak's test, Mann–Whitney and Wilcoxon signed rank test were carried out using GraphPad prism 7. Graphs are shown as mean ± standard deviation (sd) with individual patient's data points shown. PSA velocity was calculated using the online STAMPEDE calculation tools [3].

Statistical analysis of metabolite levels measured over multiple time points were conducted using the R Statistical Environment, v3.6.3 [18], the Kolmogorov–Smirnov test for normality, Levene's test for homoscedasticity and the linear mixed effects model implemented in the nlme package [19]. Metabolites found to be altered (false discovery rate <0.05, after adjustment for multiple testing) with ‘time’ during study across all patients were determined by analysing changes of the levels of individual metabolites over time during the study period. Differentially detected metabolites across the two patient groups with different PSA velocities were determined by absolute changes in their levels and/or the direction (pattern) of changes over time (i.e., increasing or decreasing).

### Inclusion of Recruited Patients for Evaluation

A recruited patient was classified as non‐evaluable if: (i) they did not complete 6 weeks of atorvastatin medication and had neither a drop of ≥50% in PSA levels from baseline nor PSA progression, or (ii) only three PSA measurements were available following the start of atorvastatin study medication and none of these corresponded to a ≥50% drop from baseline or PSA progression.

It was clarified for final analysis that patients taking at least 80% of the total 6 weeks of treatment were considered to have completed treatment.

Eighteen patients consented to participate in the study, four of whom were ineligible and did not start study treatment. Of the 14 patients registered for the study, two had taken less than the required 80% of the study treatment to be deemed evaluable. A total of 12 patients were therefore evaluable (Table 1).

**Table 1:** Details of evaluable patients.

Patient ID	Age at diagnosis, years	Gleason score at diagnosis	Age at recruitment, years	PSA at recruitment	TNM at diagnosis	TNM at progression	BMI, kg/m^2^	Weight, kg	Weeks of statin treatment	PSA at 6 weeks
2	67	9	70	3.7	T3a N0 M1	T3a N0 M1	35.4	112.9	6	6.1
5	76	9	80	10.9	T3b N1 M0	T3b N1 M0	24.7	73.8	6	13.9
6	63	9	70	2.5	T3a N0 M1	T3a NX M1	21.7	57.2	6	3.1
7	64	7	71	3.1	T3b N1 M0	T3b N1 M0	25.9	69.4	6	1.4
8	69	9	73	7.8	T4b N1 M1	T4b N1 M1	33	87.9	6	8.3
9	67	8	73	2.1	T4 N1 M0	T4 N1 M1	27.2	75.9	6	3.1
10	57	6	65	11.1	T2 NX MX	N/A	30	91.4	6	15.1
12	64	9	76	9.4	T3a N0 M0	T3a N1 M1	24.6	77.2	6	16.1
15	51	7	59	3.6	T3b N0 M0	T3b N0 M0	34.7	95.1	6	23.1
16	78	7	86	5.9	T3 N0 MO	Tx Nx M1	N/A	N/A	6	5.1
17	64	9	74	19.7	T3a N0 M0	Tx Nx0 M0	24.9	70.3	6	23
18	77	7	80	1.6	T3b N0 MX	T3b N0 M1	25.1	65.2	6	4.2

BMI, body mass index; N/A, data not available.

Among the 12 evaluable patients, three patients received prior docetaxel chemotherapy, namely, Patients #9, 10 and 18, with Patient #10 also receiving abiraterone.

Methods used for the ‘Metabolomic Study’ can be found in Appendix S1.

### Ethical Approval

The trial was approved by NHS Greater Glasgow and Clyde/University of Glasgow (GN14ON621). It was performed according to the Research Governance Framework for Health and Community Care (Second edition; 2006) and the Medicines for Human Use (Clinical Trials) Regulations, 2004 SI 2004:1031 and World Medical Association Declaration of Helsinki Ethical Principles for Medical Research Involving Human Subjects 1964. All patients provided written informed consent.

## Results

### SPECTRE Trial: Testing the Effect of Combined ADT and Atorvastatin on Prostate Cancer Disease Progression

The SPECTRE trial was designed as a proof‐of‐concept, single‐arm, two‐stage Phase II trial in patients with early evidence of CRPC, testing the impact of combined ADT and atorvastatin on disease progression (Fig. 1). Atorvastatin was selected as the statin of choice as it had been shown to be more effective at lowering cholesterol than other statins [20]. The primary study objective was to explore the impact of atorvastatin on CRPC using PSA response, defined as a ≥50% reduction from baseline, as a surrogate biomarker. Among the 12 evaluable patients for the scheduled interim analysis (Table 1; see ‘Inclusion of recruited patients for evaluation’ in Patients and Methods section), one patient satisfied the primary endpoint with ≥50% reduction in PSA levels, therefore, as defined in the study design, the study was closed to further recruitment.

**Fig. 2:**
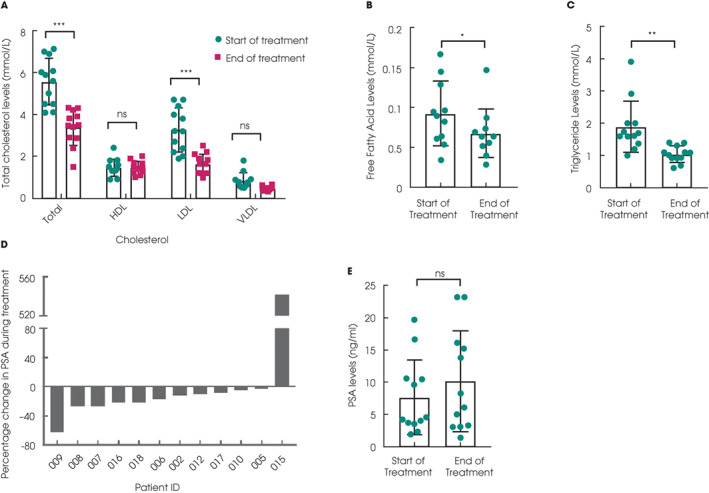
(**A**) Cholesterol levels as indicated for each informative patient (*n* = 12; ***P < 0.001, ns = not significant; two‐way ANOVA with Sidak's test; mean values ± SD are shown). Free fatty acid (**B**; *n* = 11) and triglyceride (**C**; *n* = 12) levels at the start and end of treatment for each patient (*P < 0.01, **P < 0.001; Wilcoxon signed rank test; mean values ± SD are shown). (**D**) Waterfall plot showing the maximal percentage change in PSA levels for each patient that occurs at any point after treatment start (*n* = 12). (**E**) PSA (ng/mL) levels as indicated at the start and end of treatment for each patient (*n* = 12; ns = not significant; Wilcoxon signed rank test; mean values ± SD are shown).

As expected, atorvastatin was well tolerated, with minimal reported adverse effects (Table S1). Consistent with its licensed effects, in all 12 evaluable patients, total and low‐density lipoprotein cholesterol levels significantly dropped after statin treatment, while high‐density and very‐low‐density cholesterol levels were not significantly altered (Fig. 2A, Fig. S1A). Serum levels of FFAs (Fig. 2B, Fig. S1B) and triglycerides (Fig. 2C, Fig. S1C) were also suppressed after statin treatment, albeit less dramatically than changes seen for cholesterol levels.

### Patients Can Be Stratified into Two Groups According to PSA Velocity

**Fig. 3:**
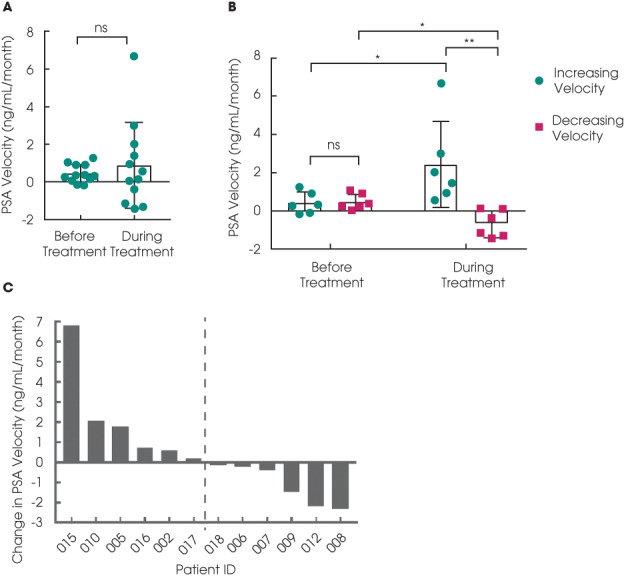
(**A**) PSA velocity for each patient before and during treatment (*n* = 12; ns = not significant; Wilcoxon signed rank test; mean values ± SD are shown). (**B**) Patients stratified into groups of those with increasing or decreasing PSA velocity comparing before and during treatment (*n* = 6 per group; *P < 0.05, **P < 0.005; Mann–Whitney test; mean values ± SD are shown). (**C**) Waterfall plot showing the change in PSA velocity comparing before and during treatment (*n* = 12). The dotted line separates the patients with increasing velocity (left) and those with decreasing velocity (right).

One of the 12 evaluable patients reported >50% of PSA response (8.3%, 80% CI 2.5%, 24.2%). The profile of changes in PSA levels after statin treatment for individual patients is presented as a waterfall plot showing the maximum (percentage and absolute) change during the course of treatment (Fig. 2D, Fig. S1A), with the majority (*n* = 11) of the 12 evaluable cases recording a decrease in PSA levels. Overall, there was no significant change in PSA from the start to the end of treatment (Fig. 2E, Fig. S1B). We then analysed PSA velocities as an exploratory endpoint [3]. For the 12 patients as a group, their PSA velocities did not change significantly after statin treatment (Fig. 3A). However, comparing their respective PSA velocities before and during treatment, the patients can be stratified into two groups, with decreased or increasing PSA velocities, respectively (Fig. 3B,C). Six of the 12 evaluable patients had rising PSA levels with increasing PSA velocities (2.44 ± 2.24 mg/mL/month), contrasting with the remaining six patients showing either stable or falling PSA levels (−0.67 ± 0.72 mg/mL/month; *P* < 0.005, Mann–Whitney test).

We found no evidence of a difference in means between the two PSA velocity groups for any of our five continuous clinical variables, namely, age (at diagnosis and recruitment), PSA levels, body mass index and weight (see Supplementary Information). There was also no association between Gleason score and PSA velocity group.

### Altered Tryptophan Metabolism Was Associated with Tumour Response to Statin Treatment

As statin treatment is thought to result in alterations in the cholesterol and related metabolic pathways, we compared the metabolic profiles of serial blood samples from our patients with diverging PSA velocities. A total of 2551 compounds/features across all samples were detected, with 973 compounds/features passing quality control and taken forward for correlative analysis with patient data. A total of 15 metabolites were identified to have adjusted *P* values <0.05, with five compounds shared among the 11 evaluable cases (serum samples were not available for Patient 015) and 10 metabolites differentially altered between patients with increasing and decreasing velocities after statin treatment.

Six of the 10 observed differentiating metabolites between patients with rising or stabilized PSA velocity after statin treatment were generated by tryptophan (Table S2): tryptophan; tryptophan's C13 naturally occurring isotope; two insource generated fragment ions; and two additional adduct ions. Running a tryptophan standard on the liquid chromatography mass spectrometry system, we observed the six detected peaks (namely, rows 3–8) mapping corresponding peaks generated by the tryptophan standard, thus confirming their identity to be related to tryptophan (Fig. S1). Manual curation revealed four key metabolites differentially detected in patients with decreased or increasing PSA velocities, with tryptophan being the dominant differentially detected peaks (Table 2). The identity of the other three metabolites could not be definitively assigned. By contrast, all five altered metabolites shared across the 12 evaluable patients did not pass manual curation assessment (Table 3), with low area under the curve and potential interaction with noise signals. Three of these five peaks were phenylalanine‐related/ fragments (Table 3).

**Table 2:** Summary of data from manual curation of 15 peaks found to be significant on ANOVA analysis.

Peak	Metabolite ID	Predicted formula	Molecular weight	RT [min]	Max. detected area	Adjusted *P* values
2	TBD	C_10_ H_16_ N_2_ O_2_	196.1212	3.061	1529163.177	0.038
4	Tryptophan	C_11_ H_12_ N_2_ O_2_	204.0899	5.128	1 739 353 326	0.036
9	TBD	C_6_ H_20_ N_7_ O_3_ PS_4_	397.02477	7.416	1375845.419	0.031
10	TBD	C_9_ H_16_ N_2_	152.13138	19.84	1722233.981	7.3E‐05

The assigned peak number corresponds with respective peak identities prior to manual curation (Table S2).

**Table 3:** Summary of commonly altered metabolites during statin treatment.

Peak	Molecular weight	RT, min	Adjusted *P* values
A	106.04182	3.579	0.008
B	495.33218	2.474	0.028
C	119.07346	3.574	0.039
D	102.04692	3.573	0.039
E	300.10477	8.183	0.039
F	165.07898	3.577	0.080

Three of the five significant peaks (A–E), namely peaks A, C and D (shaded), correspond to ions thought to be related to phenylalanine, which is represented as peak F. The main and most intense peak for phenylalanine (peak F) was clearly detected but not found to be significantly altered during the treatment period. Peak F is included in the table for reference. RT (in minutes), retention time.

## Discussion

The SPECTRE trial was the first clinical trial to directly study atorvastatin as a treatment in clinical prostate cancer. Our study is highly timely as there is mounting evidence to suggest evidence of improved patient outcome associated with statin use in advanced prostate cancer, as shown by a recent systematic review and meta‐analysis of cohort studies (https://meetings.asco.org/abstracts‐presentations/205381). Consistent with our findings, a recently published window‐of‐opportunity study of preoperative fluvastatin in localized prostate cancer [21] also revealed evidence of tumour cell kill via apoptosis (assayed by immunoreactivity of cleaved Caspase‐3), where the median treatment period of 49 days (or 7 weeks) is highly comparable to the treatment period in the SPECTRE study. It is worth noting that atorvastatin is one of the most potent cholesterol‐lowering agents, approximately three times more potent than fluvastatin at the same dose [20].

The magnitude of observed tumour response in our study was subtle, with ‘flattening’ of PSA velocity following a short course of statin treatment observed in six of 12 evaluable patients. Our primary objective was to investigate whether we could detect any evidence of statin‐mediated tumoral effects based on PSA measurements. In hindsight, for a proof‐of‐concept study, the criteria of tumour response with a ≥50% drop in serum PSA levels can be considered overambitious, given the fact that, at a population level, the association between statin treatment and favourable patient outcome typically requires a sustained period of statin treatment. Nonetheless, despite the short duration of treatment, our data on percentage fall in serial PSA measurements as well as PSA velocity are supportive of previous observations of favourable patient outcome with the use of statins in population‐based studies. We have designed the SPECTRE study to specifically investigate the potential benefits of adding statins to ADT in patients with CRPC. In future studies, it will be necessary to study patients treated with docetaxel chemotherapy or other novel androgen receptor pathway inhibitors along with ADT. We were also not able to determine which patients may benefit from the combined statin and hormone therapy, so further study is warranted to potentially establish the criteria for patients to benefit. Furthermore, the detection of differential serum tryptophan levels between patients with rising or stabilized PSA velocity raises the possibility that statin treatment functionally impacts the tumour biology of CRPC.

To formally test if altered tumoral cholesterol metabolism resulted from statin treatment, direct analysis of tumour materials is required. Paired prostatic biopsies (before and during treatment) were offered for optional patient consent. Unfortunately, not enough prostate biopsy samples were obtained. In accordance with our study design, recruitment was closed following the scheduled interim analysis as the criteria of ≥2 patients achieving >50% PSA drop was not reached. Another limitation of this study was the relative early stage of CRPC among recruited patients, with relatively low serum PSA levels at recruitment. For patients with more significant disease, there was a tendency to proceed with licensed treatment rather than participating in a proof‐of‐concept study. For future study with statins, it would be of interest to consider a combination approach with second‐generation antiandrogen agents or with chemotherapy.

Tryptophan metabolism can influence a wide range of pathophysiological conditions including cancer [22]. Consistent with our findings, altered serum tryptophan levels have been reported in patients at risk of CRPC [23]. Of note, statins can also alter tryptophan metabolism [24], either directly through their effects on cholesterol metabolism or indirectly via other targets in cytokine‐producing immune cells. A recent report highlighted the importance of the potential association between elevated circulating sphingolipids with genetic aberrations in metastatic CRPC, such as *AR*, *TP53* and *RB1*, and that a biomarker signature combining lipid and genetic abnormalities may be predictive of worse prognosis [25].

In summary, our proof‐of‐concept study revealed data suggestive of stabilization of CRPC within a 6‐week study period. Given the current treatment options for second‐ and third‐line androgen receptor pathway inhibitors, we found that, for patients with advanced CRPC, both oncologists and patients favoured the commencement of standard of care treatment rather than taking part in the SPECTRE study. Hence, the design of future studies may incorporate the use of statins with one of the androgen receptor pathway inhibitors, requiring the recruitment of large patient numbers with extended duration of statin treatment to fully test the long‐term anti‐cancer effects of statins.

## Disclosures of Interest

The authors declare no disclosures of interest.

**Abbreviations**
ADT: androgen deprivation therapy
CRPC: castration‐resistant prostate cancer
FFA: free fatty acid

## Supporting information


**Appendix S1.** Supplementary Information on patients, methods and data analysis, and supplementary.

## Data Availability

The data underlying this article will be shared on reasonable request to the corresponding author.
