# Supplementary material for: Phase II proof‐of‐concept study of atorvastatin in castration‐resistant prostate cancer
Source: BJU Int. 2022 Aug 12;131(2):236–43. doi: 10.1111/bju.15851 (PMC10087532; doi:10.1111/bju.15851)

Supplementary Information on patients, methods and data analysis, and supplementary tables.

Metabolomic study

Serum samples were diluted 50-fold with ice cold extraction solvent (50% methanol, 30% acetonitrile, 20% water) and vortexed. Samples were centrifuged at 16,100 x g for 10 minutes at 0-4°C. The supernatants were transferred to glass HPLC vials and kept at -75°C prior to LC-MS analysis

A Q Exactive plus Orbitrap mass spectrometer coupled to an Ultimate 3000 HPLC system (Thermo Scientific, Waltham, MA, USA) was used for the analysis as previously described (1). Extracts were separated using a ZIC-pHILIC column (SeQuant, 150 x 2.1 mm, 5 µm, Merck KGaA, Darmstadt, Germany) held at a column temperature of 45°C. Initial mobile phase consisted of 20% 20 mM ammonium carbonate, pH 9.2, and 80% acetonitrile, metabolites were separated over a 15-minute gradient decreasing the acetonitrile content to 20%, at a flow rate of 200 µL/min. A mass range of 75-1000 m/z at a resolution of 70,000 (at 200m/z) with polarity switching was recorded across the LC-MS run. A pooled sample (1:1 pool of all sample extracts) was prepared and analysed throughout acquisition at multiple times as a QC control. The same pool was analysed separately in positive and negative single ionization mode using data dependent fragmentation (ddMS2) to assist with metabolite identification. Data were acquired with Xcalibur and processed using Compound Discoverer v3.1 software (Thermo Scientific).

Retention times (RT) were aligned across all raw files (maximum shift 2 min, mass tolerance 5 ppm). Unknown compound detection (minimum peak intensity 5e5, RT > 2 min) and grouping of compound adducts was carried out (mass tolerance 5 ppm, RT tolerance 0.7

min). Missing values were filled using the software's Fill Gap feature (mass tolerance 5 ppm, S/N tolerance 1.5). The raw result table was further filtered, only compounds present in 50% of the pooled QC runs with an Area RSD <30% was taken forward, of these only compounds with a successful Predict formula compositions (Mass Tolerance 5 ppm, minimum spectral fit and pattern coverage of 30% and 90% respectively) were further analysed. Following statistical analyses (repeated measures ANOVA analysis, see statistical analysis below), candidate peaks were screened manually for good and consistent peak integration.

Tryptophan was successfully identified by matching the mass and retention time of observed peaks to an in-house database generated using metabolite standards (mass tolerance 5 ppm, RT tolerance 0.5 min). The annotation was further confirmed using mzCloud (ddMS2) database search (Precursor and fragment mass tolerance of 10 ppm, match factor threshold 50) with a MS2 spectral match generating a score of 97.7/100.

Statistical analysis of metabolite levels measured over multiple time points were conducted using the R Statistical Environment, v3.6.3 (2), the Kolmogorov-Smirnov test for normality, Levene's test for homoscedasticity and the linear mixed effects model implemented in the nlme package (3). Metabolites found to be altered (FDR<0.05, after adjustment for multiple testing) with 'time' during study across all patients were determined by analysing changes of the levels of individual metabolites over time during the study period. Differentially detected metabolites across the two patient groups with different PSA velocities were determined by absolute changes in their levels and/or the direction (pattern) of changes over time (i.e. increasing or decreasing).

Supplementary results

<u>Reported adverse events</u>

There were no severe unexpected adverse events attributed to study treatment. There were two serious adverse events which were considered unrelated to the study interventions (one transient ischemic attack and one episode of syncope related to cardiac dysrhythmia) (Table S1).

<u>Analysis of clinical parameters</u>

Clinical parameters (at diagnosis - tumour Gleason score, age; at recruitment – age, PSA levels, BMI and weight) were analysed against the two observed PSA velocity sub-groups. Age, PSA levels, BMI and weight were considered as continuous data.

Gleason score at diagnosis

Since Gleason score is categorical, the data can be expressed in the following contingency table:

	Group	
Gleason score at diagnosis	Reduction in PSA velocity	No reduction in PSA velocity
6	1	0
7	2	2
8	0	1
9	3	3

Fisher's Exact Test was then used to test for an association between Gleason score at diagnosis and Group. No evidence of an association was found ($p = 1.0$).

Age at diagnosis

The Shapiro-Wilk normality test indicated that there is no evidence of non-normality in the data, for either group. We therefore proceeded to perform a t-test to test for a difference in the mean age at diagnosis between the two groups. We found no evidence of a difference in the mean age at diagnosis between the two groups ($p = 0.7133$). Patients with a reduction in PSA velocity were estimated to be, on average, 1.8 years older (95% confidence interval: (-9.4, 13.1) years older) at diagnosis than patients without a reduction in PSA velocity.

Age at recruitment

The Shapiro-Wilk normality test indicated that there is no evidence of non-normality in the data, for either group. We therefore proceeded to perform a t-test to test for a difference in the mean age at recruitment between the two groups. We found no evidence of a difference in the mean age at recruitment between the two groups ($p = 0.7379$). Patients with a reduction in PSA velocity were estimated to be, on average, 1.5 years older (95% confidence interval: (-8.9, 11.9) years older) at recruitment than patients without a reduction in PSA velocity.

PSA at recruitment

The Shapiro-Wilk normality test indicated that there is no evidence of non-normality in the data, for either group. We therefore proceeded to perform a t-test to test for a difference in the mean PSA at recruitment between the two groups. We found no evidence of a difference in the mean PSA at recruitment between the two groups ($p = 0.1373$). Patients with a

reduction in PSA velocity were estimated to have, on average, a PSA at recruitment that is 4.73 units lower (95% confidence interval: (-1.89, 11.36) units lower) than patients without a reduction in PSA velocity.

BMI

The Shapiro-Wilk normality test indicated that there is no evidence of non-normality in the data, for either group. We therefore proceeded to perform a t-test to test for a difference in mean BMI between the two groups. We found no evidence of a difference in mean BMI between the two groups ($p = 0.2224$). Patients with a reduction in PSA velocity were estimated to have, on average, a BMI that is 3.69 units lower (95% confidence interval: (-2.80, 10.18) units lower) than patients without a reduction in PSA velocity.

Weight

The Shapiro-Wilk normality test indicated that there is no evidence of non-normality in the data, for either group. We therefore proceeded to perform a t-test to test for a difference in mean weight between the two groups. We found no evidence of a difference in mean weight between the two groups ($p = 0.1077$). Patients with a reduction in PSA velocity were estimated to be, on average, 16.57 kg lighter (95% confidence interval: (-4.79, 37.93) kg lighter) than patients without a reduction in PSA velocity.

Manual curation of the compounds/features from liquid-chromatography mass-spectrometry analysis highlighted by repeated measures ANOVA analysis to be significant

Of the 2551 compounds/features detected in the SPECTRE serial blood samples, 973 compounds/features passed quality control and were included in a repeated measures ANOVA analysis. A total of 15 metabolites were identified to have adjusted p value <0.05, with five compounds shared among the 11 evaluable cases (serum samples were not available for patient 015) and 10 metabolites differentially altered between patients with increasing and decreasing PSA velocity following statin treatment.

Six of the ten observed differentiating metabolites between patients with increasing or decreasing PSA velocity following statin treatment were generated by tryptophan (Figure S3, Table S2): tryptophan, tryptophan's C13 naturally occurring isotope, two insource generated fragment ions and two additional adduct ions. Running a tryptophan standard on the liquid chromatography mass spectrometry system, we observed all six detected peaks (namely rows 3-8) mapping corresponding peaks generated by the tryptophan standard, thus confirming their identity to be related to tryptophan. Of the five altered metabolites shared across the 12 evaluable patients (Table S3), none of the peaks passed manual curation assessment, with low area under curve and potential interaction with noise signals. Three of these 5 peaks were phenylalanine related/ fragments.

References

1. Mackay GM, Zheng L, van den Broek NJ, Gottlieb E. Analysis of Cell Metabolism Using LC-MS and Isotope Tracers. Methods Enzymol. 2015;561:171-96.
2. Team RC. R: A Language and Environment for Statistical Computing. https://www.R-project.org/. 2020.
3. Pinheiro J, Bates D, DebRoy S, Sarkar D, Team RC. nlme: Linear and Nonlinear Mixed Effects Models. R package version 3.1-150, https://CRAN.R-project.org/package=nlme. 2020.

Supplementary Figure Legends

Figure S1.

A-C. Serum levels for each patient over the course of atorvastatin treatment of **A.** Cholesterol (n=12); **B.** Free fatty acids (n=11); **C.** Triglycerides (n=12). Each patient is represented by a different colour of line as indicated.

Figure S2.

A. Waterfall plot showing the maximal absolute change in PSA levels that occurs at any point after treatment start (n=12).

B. Serial PSA levels for evaluable patients (n=12). Each patient is represented by a different colour of line as indicated.

Figure S3.

Detailed analysis of six peaks to confirm their identity to be tryptophan related.

Supplementary Table.

Table S1. Serious adverse events (SAE)

SAE Reference Number	Record Number	SAE Short Name	Other Details	Grade	Outcome	Relationship to Atorvastatin
U233SAE-6-1	1	Transient ischemic attacks		1	Resolved with no sequelae	Unrelated
U233SAE-16-1	1	Syncope		1	Resolved with sequelae	Unrelated
U233SAE-16-1	2	Cardiac disorders - Other, specify	Trifascicular block and intermittent 2:1 atrio-ventricular block	3	Resolved with sequelae	Unrelated

Table S2. Differentially detected metabolites in patients with rising or stabilised PSA velocities following statins treatment

The rows referring to tryptophan-related peaks (namely rows 3-8) are presented in shade. Peaks presented in row 1 has low signal to noise ratio and removed for further consideration. RT (in minutes) = retention time; Area = area under curve for metabolite peaks. Adjusted p values from ANOVA analysis for correlation to patient response judged by rising or stabilised PSA velocities. (TBD, to be determined)

Peak	Name	Formula	Molecular Weight	RT [min]	Area	Adjusted p values
1	TBD	C5 H7 N O	97.0528	2.531	1542506.612	0.00082
2	TBD	C10 H16 N2 O2	196.1212	3.061	1529163.177	0.038
3	Tryptophan related	C20 H26 N5 O3 P	415.17719	5.024	5190192.384	0.043
4	Tryptophan	C11 H12 N2 O2	204.0899	5.128	1739353326	0.036
5	Tryptophan related	C11 H9 N O2	187.06332	5.129	1583210277	0.043
6	Tryptophan related	C8 H7 N	117.05786	5.142	14628571.2	6E-05
7	Tryptophan related	C4 H11 N7 O3	205.09311	5.144	130812026.7	0.017
8	Tryptophan related	C11 H13 Cl N2 O2	240.06702	5.237	22072540.21	0.038
9	TBD	C6 H20 N7 O3 P S4	397.02477	7.416	1375845.419	0.031
10	TBD	C9 H16 N2	152.13138	19.84	1722233.981	7.3E-05

Figure S1.

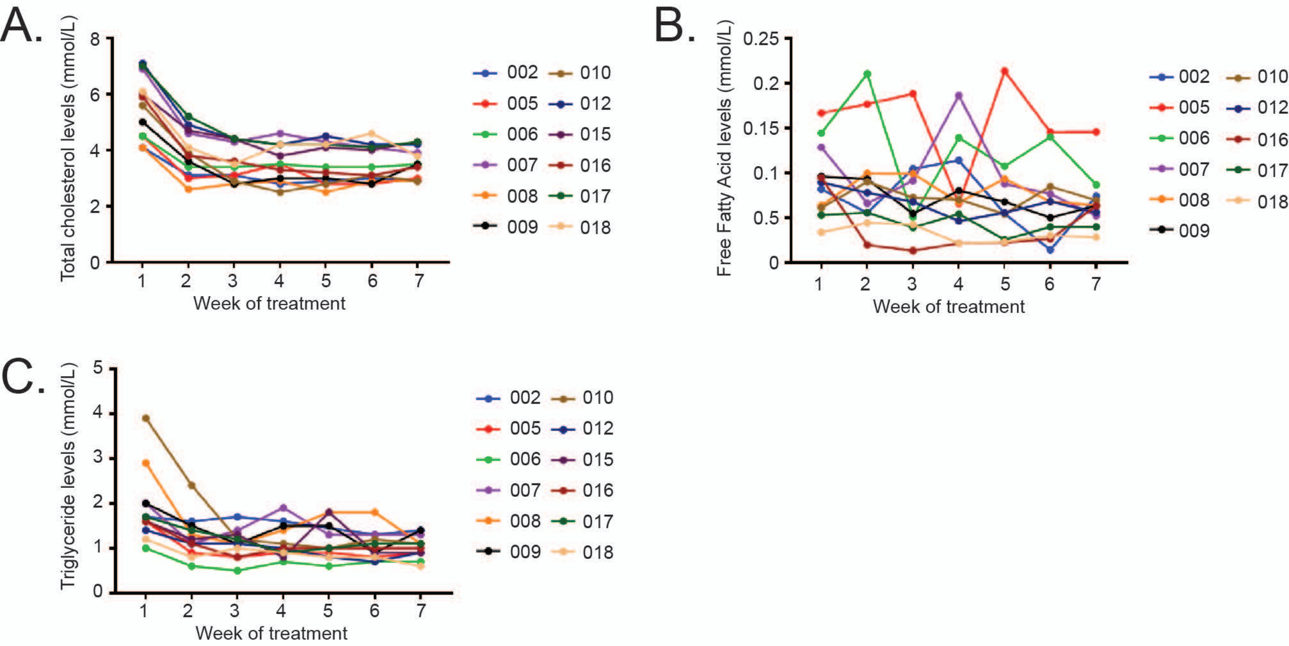
A.
Total cholesterol levels (mmol/L)
8
6
4
2
0
1 2 3 4 5 6 7
Week of treatment
002
010
005
012
006
015
007
016
008
017
009
018
B.
Free Fatty Acid levels (mmol/L)
0.25
0.2
0.15
0.1
0.5
0
1 2 3 4 5 6 7
Week of treatment
002
010
005
012
006
016
007
017
008
018
009
C.
Triglyceride levels (mmol/L)
5
4
3
2
1
0
1 2 3 4 5 6 7
Week of treatment
002
010
005
012
006
015
007
016
008
017
009
018

Figure S2.

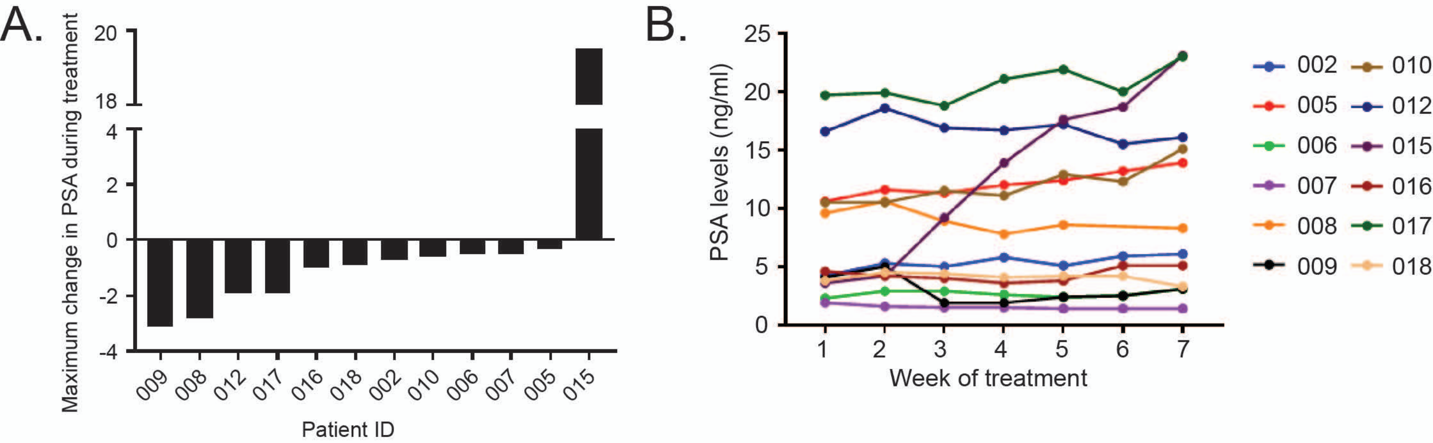
A.
Maximum change in PSA during treatment
20
18
4
2
0
-2
-4
009
008
012
017
016
018
002
010
006
007
005
015
Patient ID
B.
PSA levels (ng/ml)
25
20
15
10
5
0
1
2
3
4
5
6
7
Week of treatment
002
005
006
007
008
009
010
012
015
016
017
018

Figure S3.

Tryptophan Standard

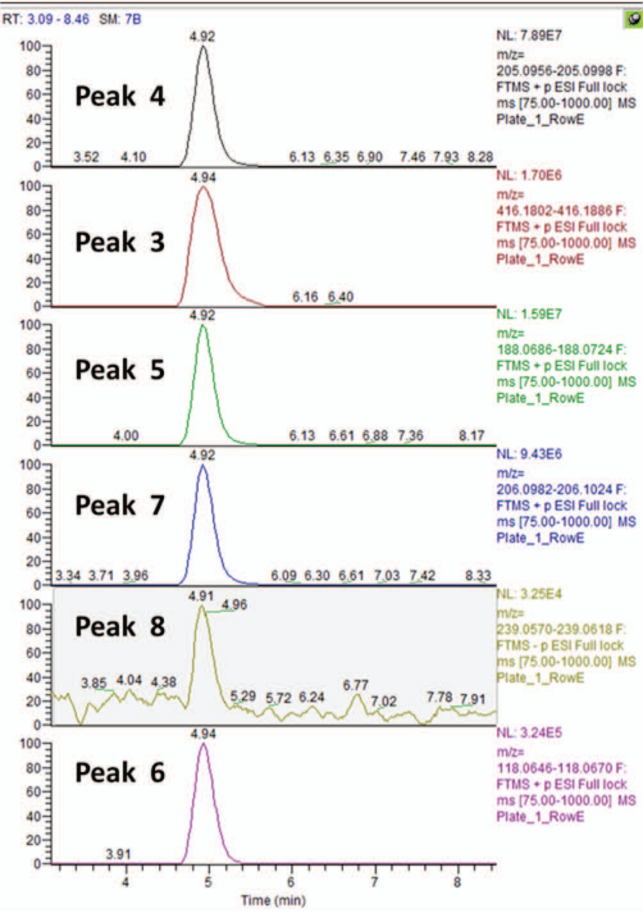

Supplement: Supplementary file 1 — Appendix S1. Supplementary Information on patients, methods and data analysis, and supplementary. [file BJU-131-236-s001.pdf]
